# Efficacy of indigenous bacterial antagonists from the anthosphere of *Macadamia integrifolia* in controlling Cladosporium raceme blight

**DOI:** 10.1007/s11274-026-04997-9

**Published:** 2026-05-02

**Authors:** Marcos Giovane Pedroza de Abreu, Luana Laurindo de Melo, Vanessa Rafaela de Carvalho, Leonardo Massaharu Moriya, Sonia Claudia do Nascimento de Queiroz, Bernardo de Almeida Halfeld-Vieira

**Affiliations:** 1 Faculdade de Ciências Agronômicas, Universidade Estadual Paulista, Botucatu, Brazil; 2 QueenNut Macadamia, Dois Córregos, Brazil; 3 Embrapa Meio Ambiente, Jaguariúna, Brazil

**Keywords:** Anthosphere, *Bacillus subtilis*, *Biological control*, *Cladosporium xanthochromaticum*, Raceme blight, *Serratia ureilytica*

## Abstract

Floral diseases of macadamia, such as raceme blight, are among the main limiting factors for crop production, and the low availability of registered products for phytosanitary management further aggravates this challenge. In this context, biocontrol agents emerge as a sustainable and effective alternative for integrated disease management. This study aimed to select antagonistic bacteria for the control of raceme blight in macadamia, caused by *Cladosporium xanthochromaticum*. For this purpose, bacteria were isolated from macadamia racemes, identified, and tested against the phytopathogen in both in vitro and in vivo experiments (racemes and field plants). Their mechanisms of action and compatibility with pesticides registered for the crop were also evaluated. Strains of *Serratia ureilytica* and *Bacillus subtilis* proved effective in controlling the disease in the field. *S. ureilytica* reduced the incidence of *C. xanthochromaticum* by 27.04% to 35.38% and its sporulation by 52.04% to 52.65%. Meanwhile, *B. subtilis* was effective in reducing sporulation in racemes by 16.25% to 26.08%. Both strains demonstrated inhibitory activity through volatile and non-volatile compounds, as well as competition for nutrients. The strains were compatible with all pesticides tested, except copper hydroxide, to which *S. ureilytica* was sensitive. The results indicate that the selected strains can be incorporated into the integrated disease management of macadamia, contributing to the reduced reliance on chemical pesticides.

**Supplementary Information:**

The online version contains supplementary material available at 10.1007/s11274-026-04997-9.

## Introduction

Macadamia (*Macadamia integrifolia*) is a plant native to Australia whose cultivation has expanded to several countries. Currently, South Africa, Australia, and China are the largest producers of macadamia nuts, while Brazil ranks ninth among the world’s largest macadamia nut consumer markets (INC, 2024). The steady increase in consumption over the last five years has encouraged Brazilian producers to expand cultivation areas to meet this rising demand.

However, a major factor limiting productivity is the occurrence of pests and diseases, especially those affecting fruit development. In Australia, during the 2023/2024 season, floral diseases were a significant constraint on productivity (Bright 2024). In Brazil, raceme blight (*Cladosporium xanthochromaticum*) is one of the main phytosanitary problems affecting macadamia (Silva et al. 2024). By affecting reproductive organs, this disease can lead to low fruit production and yield losses of up to 80% (Prasannath et al., 2022a). Losses result not only from the death of flowers that could have produced fruits, but also from reduced cross pollination of healthy flowers (Howlett et al. 2015; Trueman 2013).

A limitation in disease management is the lack of registered pesticides for controlling macadamia raceme blight in Brazil. Current management practices rely mainly on cultural strategies, such as pruning the canopy to improve flower aeration and removing racemes remaining from previous seasons (Bright 2024; Prasannath et al. 2022a, b). The scarcity of options often leads producers to use products registered for other crops with similar problems.

Biological control, therefore, represents a viable approach for disease management, as it is recognized as an environmentally low-impact practice. Biocontrol agents can suppress phytopathogens through multiple mechanisms, including antibiosis via volatile and non-volatile compounds, competition for space or nutrients, induction of host defense mechanisms, and parasitism (Köhl et al. 2019). The use of biological control of pathogens has increased, reducing the use of chemical pesticides while contributing to agroecosystem balance. This is particularly relevant in macadamia, a crop that relies heavily on natural bee pollination, since repeated fungicide applications on floral tissues may increase the risk of pollinator contamination and associated negative effects on pollination services.

Although several studies report the potential of selected antagonists in controlling both foliar and soilborne pathogens, biological control in the anthosphere has been scarcely explored (Compant et al. 2019; Kim et al. 2023; Temmermans et al. 2025).

The present study aimed to select antagonistic bacteria capable of controlling macadamia raceme blight and to elucidate their mechanisms of action. To this end, assays were conducted to: (1) isolate and identify antagonistic bacteria from the anthosphere; (2) evaluate their efficacy in controlling raceme blight both on excised racemes and under field conditions; and (3) determine the mechanisms involved in pathogen suppression.

## Materials and methods

### Source of the phytopathogen and bacterial isolates

The *Cladosporium xanthochromaticum* CMAA1835 strain used in this study was obtained from the Collection of Microorganisms of Agricultural and Environmental Importance (CMAA) of Embrapa Meio Ambiente, Jaguariúna, São Paulo, Brazil (Silva et al. 2024).

Racemes for the isolation of antagonists were collected in 2021 from the commercial orchard of QueenNut Macadamia, located in Dois Córregos, São Paulo, Brazil (22°19’08"S 48°22’01"W). Samples were collected from the canopy periphery of 10 trees, at an average height of 1 to 2 m above the ground, placed in paper bags, and transported to the laboratory.

For the isolations, the vegetative whorls (rachis, peduncle, and petals) and reproductive whorls (stamens and carpels) of the collected racemes were processed individually. To obtain the total bacterial population, the plant structures were placed in 150 mL Erlenmeyer flasks containing 50 mL of sterile saline solution (0.85% NaCl) with 0.3% Tween 80 [Sigma, Germany] and shaken at 300 rpm for 20 min (Halfeld-Vieira et al., 2004). Serial dilutions of the extract, up to 1:10,000, were prepared, and 100 µL of each dilution was plated in duplicate onto Petri dishes containing 523 culture medium (Kado and Heskett 1970). Aliquots were spread using a Drigalski spatula and were incubated at 25 °C. Pure cultures, preferably morphologically distinct, were obtained from plates with isolated colonies, transferred to test tubes containing 523 medium, and stored at 4 °C.

### Initial screening

Initial screening was performed through in vitro antibiosis assays and by evaluating the antagonists’ effects on the incidence and sporulation of *C. xanthochromaticum* CMAA1835 on detached racemes of the IAC4-12B cultivar, in order to select the most effective antagonists.

The antagonists were cultured on 523 medium at 25 °C for up to 48 h and inoculated onto PDA (Kasvi, Curitiba, PR, Brazil). 39 g/L, containing dehydrated potato infusion, dextrose, and agar, pH 5.6 ± 0.2 at 25 °C, prepared according to the manufacturer’s instructions.) in two orthogonal lines from the edge of Petri dishes, stopping 2.0 cm from the center. A 0.5 cm diameter mycelial disc, collected from the margins of a 14-day-old actively growing colony of *C. xanthochromaticum* using a sterilized cork borer, was placed in the uninoculated central region. Three replicates were used, each consisting of a single plate. Plates were incubated at 23 °C for up to 5 days. The bacterial isolates were evaluated based on their qualitative inhibitory capacity, defined by the formation of distinct inhibition zones (clear areas between the bacterial colony and the fungal mycelium). Only isolates that consistently exhibited a visible inhibition zone were pre-selected and subjected to detached raceme assays at phenological stage 3 (anthesis) (Prasannath et al. 2022a, b).

For this assay, *C. xanthochromaticum* CMAA1835 colonies were cultured on PDA for 14 days at 23 °C, and a conidial suspension (10³ conidia/mL) was prepared. Pre-selected bacterial isolates were cultured in 523 medium for 24 h at 25 °C, and a cell suspension was prepared in 0.85% NaCl with 0.03% Tween 80, adjusted to A₅₄₀= 0.3 (Halfeld-Vieira et al., 2004).

Detached racemes were sprayed with bacterial suspensions until run-off and maintained at 23 °C for 24 h. Subsequently, the racemes were sprayed until run-off with a *C. xanthochromaticum* suspension and incubated in a moist chamber, which consisted of plastic bags moistened with sterile distilled water to maintain high humidity. Controls included racemes sprayed with sterile distilled water (uninoculated control) and racemes sprayed only with the *C. xanthochromaticum* suspension (pathogen-only control). The experiment followed a completely randomized design with four replicates per treatment, where each raceme represented one experimental unit.

Disease incidence was assessed four days post-inoculation, a time point that corresponds to the pathogen’s incubation period when the first characteristic symptoms become clearly visible, by observing necrosis which, as the disease progressed, culminated in the appearance of initially olive-green and later brownish-gray superficial fungal growth on the flowers, as well as visible signs of fungal colonization, on 25 flowers per raceme per replicate, and was expressed as the percentage of symptomatic flowers within this sample. To determine the antagonists’ effect on sporulation, five inflorescences per replicate were placed in test tubes with 5 mL sterile distilled water, vortexed, and spore concentration was determined using a hemocytometer.

Experiments were conducted in a completely randomized design. Incidence data were arcsine-transformed and sporulation data log-transformed to meet assumptions of normality and homogeneity of variance. Analysis of variance (ANOVA) was performed, and means were compared using the Scott-Knott test (*p* < 0.05) in R software version 4.3.2 (R Core Team, Vienna, Austria).

### Antagonists identification

The identification of selected bacterial antagonists was performed via 16 S rRNA sequencing and multilocus sequence analysis (MLSA). Genomic DNA was extracted using Q-Extract DNA Extraction Solution (Neobio, Taboão da Serra, SP, Brazil) combined with proteinase K (Sigma-Aldrich, St. Louis, MO, USA) (20 mg/mL) at 95 °C for 20 min. Amplification of the 16 S rRNA gene was performed with primers 16S08F (GYCCADACWCCTACGG) and 16S08R (CACGAGCTGACGAC) (Arruda et al. 2017). PCR reactions (25 µL) consisted of 12.5 µL Taq DNA polymerase (Neobio, Taboão da Serra, SP, Brazil), 7.5 µL Milli-Q water, 1 µL of each primer, and 3 µL DNA template with initial denaturation at 94 °C for 7 min; 30 cycles of 94 °C for 45 s, 50 °C for 30 s, 72 °C for 60 s; final extension at 72 °C for 10 min (Arruda et al. 2017). Amplification products were visualized on 1% agarose gel using a UV transilluminator (Major Science, New Taipei City, Taiwan) and a 100 bp molecular marker (Norgen Biotek, Thorold, ON, Canada), purified using magnetic beads (Agencourt, Beckman Coulter, Brea, CA, USA), and quantified with a NanoDrop spectrophotometer (Thermo Fisher Scientific, Waltham, MA, USA).

For multilocus sequence typing (MLST) to confirm the identity of antagonistic strains CMAA1920 and CMAA1922 and construct a phylogenetic tree, primers *gyrB* and *rpoB* (Scrascia et al. 2016) and tuf2 (Xu et al. 2023) were used. PCR conditions followed the protocols of Scrascia et al. (2016) and Xu et al. (2023). Amplified products were purified, quantified, and sent for Sanger sequencing. Sequences were compared against GenBank using BLAST and aligned with ClustalW (Thompson et al. 1994). Phylogenetic trees were generated in MEGA X using the best-fit model and bootstrap analysis (Kumar et al. 2018; Felsenstein 1985).

For isolate CMAA1920, the ability to utilize urea as the sole carbon source was tested to corroborate the species identification. Cultures were grown in basal medium (g/L: MgSO₄·7 H₂O, 0.5; KH₂PO₄, 0.5; KCl, 0.1; pH 6.5) supplemented with 50 mM glucose, 0.5 M urea, and 0.01% yeast extract (all reagents were of analytical grade). Negative controls lacked urea. Bacterial suspensions were inoculated into 20 mL of basal medium and incubated at 27 °C under agitation for 48 h. Growth was assessed visually by turbidity (Bhadra et al. 2005).

### Field assay

The field experiment was conducted in a 15-year-old macadamia orchard at the commercial site of QueenNut Macadamia, Dois Córregos, São Paulo, Brazil (22°19’08"S 48°22’01"W) during the flowering period of the 2023 agricultural year. Two trials were performed, one in June and another in August, using the two antagonists that demonstrated the highest disease control in the initial screening test. Preparation of antagonist and pathogen suspensions, as well as inoculations, were performed as described for the detached raceme assay.

Bacterial suspensions of antagonists CMAA1920 and CMAA1922 (A₅₄₀= 0.3) were sprayed until run-off onto flowers of macadamia trees (cv. IAC 4-12B) at phenological stages 2 and 3 (pre-anthesis and anthesis). Applications were performed in the early morning using a manual sprayer. Racemes treated with sterile distilled water served as the absolute control, while those treated with the commercial fungicide Supera^®^ (copper hydroxide, 537 g/kg, Mitsui & Co., Tokyo, Japan) were used as the standard for disease control. Each treatment was applied to six trees, with three racemes per phenological stage per tree (n = 18 racemes per treatment). Following the antagonist application until run-off, racemes were incubated for 24 h in a moist chamber consisting of polystyrene bags. Subsequently, the racemes were sprayed until run-off with a *C. xanthochromaticum* suspension (10^5^ conidia/mL) and re-incubated in the moist chamber for an additional 24 h. Afterward, the polystyrene bags were replaced with paper bags. Ten days post-inoculation, racemes were collected for evaluation of disease incidence and spore counting, as described in the ‘*Initial Screening*’ section.

The experiment was conducted in a completely randomized design in a factorial scheme with 18 replicates. Sporulation data from the second trial were square-root transformed to meet homogeneity assumptions. ANOVA was performed, and means were compared using Tukey’s test (*p* < 0.05) in R software version 4.3.2 (R Core Team, Vienna, Austria).

### Characterization of control mechanisms of selected antagonists

#### Nutritional similarity

Nutritional profiles based on utilization of unique carbon and nitrogen sources were determined for antagonists CMAA1920 and CMAA1922 and for the pathogen *C. xanthochromaticum* CMAA1835. Bacterial profiles were assessed using both GN2 and GP2 MicroPlates™ for each antagonist, and FF MicroPlates™ (Biolog, Inc., Hayward, CA, USA) for *C. xanthochromaticum*, following manufacturer’s instructions. Each plate contained 95 unique carbon sources and a control well. Color development in wells was measured spectrophotometrically at 590 nm every 24 h up to 72 h.

Nutritional similarity was estimated using the niche overlap index (NOI). This index was calculated to estimate the niche overlap between the antagonistic bacteria and *C. xanthochromaticum*, and vice versa, using the formula: NOI = the number of carbon sources used by both the antagonistic bacterium and the pathogen / the total number of carbon sources used by the organism being overlapped (Halfeld-Vieira et al. 2015).

Indices were calculated for individual sources and for specific substrate groups: carbohydrates, amino acids, organic acids, alcohols, amides/amines, and miscellaneous. Values above 0.9 indicated high competition for C or N sources, suggesting effective niche exclusion. Values below 0.9 indicated low competitive ability (Halfeld-Vieira et al. 2015; Wilson and Lindow 1994a, b).

#### Chitinase Production

Chitinase production by antagonists was assessed using the mineral medium of Renwick et al. (1991) with 0.08% colloidal chitin (Sigma-Aldrich, St. Louis, MO, USA) as the sole carbon source. Bacteria were inoculated at distinct points on the medium and incubated at 25 °C for 10 days, with three replicates per treatment. Chitinase activity was indicated by the presence of a transparent halo around colonies against the opaque medium (Pelzer et al. 2011).

#### β-1,3-Glucanase production

β-1,3-Glucanase activity was determined using mineral medium supplemented with 0.5% laminarin (Sigma-Aldrich, St. Louis, MO, USA) as the sole carbon source (Renwick et al. 1991). Cultures were inoculated on the medium surface and incubated at 25 °C for 10 days (three replicates). The medium surface was stained with 0.5% Congo Red solution for 90 min. It was then washed, and the enzymatic activity was indicated by a light orange halo around colonies (Pelzer et al. 2011).

#### Antibiosis via volatile compounds

Volatile antibiosis tests followed Essa Al-Rubkhi et al. (2024) with modifications. A 0.2 µL aliquot of *C. xanthochromaticum* spore suspension (10⁵ conidia/mL) was placed in the center of a 90-mm Petri dish containing PDA. In a separate 90-mm Petri dish, a 100 µL aliquot of bacterial suspension (A₅₄₀= 0.1) was spread on 523 medium. The lids were removed, and the Petri dish containing the fungal culture was inverted and placed on top of the dish containing the bacterial culture. The two dishes were then sealed together with Parafilm M (Bemis Company Inc., Neenah, WI, USA), and incubated at 23 °C for 10 days. Controls consisted of the fungal plates over uninoculated 523 medium. Inhibition (%) was calculated by measuring fungal colony diameters. Ten replicates per treatment were conducted. Data were analyzed using a t-test (*p* < 0.05) in R software version 4.3.2 (R Core Team, Vienna, Austria).

### Volatile organic compounds (VOCs) determination

#### Solid phase microextraction extraction (SPME)

Petri plates containing antagonist cultures were placed inside autoclaved glass jars (2280 ± 7 ml) sealed with septum-containing caps, and equilibrated for 1 h for headspace VOC accumulation. SPME fiber (DVB/CAR/PDMS 50/30 µm, Stableflex-Gray, Supelco) was exposed for 15 min, then desorbed in a GC-MS injector for compound separation and identification. Fiber was cleaned between injections for 10 min.

#### Instrumental analysis

VOCs were analyzed using an Agilent 7890B GC coupled to a 5977B MS. Separation was performed on a 30 m x 0.25 mm x 0.25 μm HP-5MSUI column. Injector temperature was set to 250 °C in splitless mode. Oven program was initially kept at 40 °C for 1 min, then ramped to 150 °C at 5 °C/min (1 min hold), followed by an increase to 250 °C at 10 °C/min (1 min hold). The total runtime was 35 min. Detection in GC-MS was performed using 70 eV ionization over a mass range of 50–550 amu. The ion source temperature was set at 230 °C, and the interface temperature at 150 °C. Helium was used as carrier gas at a flow of 1.2 mL/min. Deconvolution and identification were performed using Agilent MassHunter Workstation software Unknowns Analysis and NIST Tandem Mass Spectral Library v2.3.

#### Inhibition of conidial germination by cell-free filtrate

In Petri dishes, a 2-mm layer of water-agar medium containing the cell-free filtrate (CFF) was added. To obtain the cell-free filtrate (CFF), bacterial colonies were grown in 523 medium (Kado and Heskett) under constant shaking for 24 h at 25 °C, and the culture density was adjusted to A_540_ = 0.1. Cells were pelleted by centrifugation at 4,000 rpm for 10 min, and the supernatant was aseptically filtered through a 0.22-µm membrane filter (Kasvi, Curitiba, PR, Brazil) to obtain a CFF free of bacterial cells. The CFF was then mixed with water–agar medium (17 g L⁻¹ agar, maintained at 50 °C) at a final proportion of 80:20 (v/v, CFF: water–agar) to prepare the medium used in the conidial germination assays (Pellicciaro et al. 2021). A 10 µL aliquot of the *C. xanthochromaticum* conidial suspension was deposited onto the culture medium. Eight replicates were used for each treatment, along with a control treatment in which plates contained only 80% (v/v) water-agar medium without CFF. After 16 h of incubation at 23 °C, at least 100 conidia per Petri dish were evaluated by direct observation of the bottom of the plates using a microscope. Conidia were classified as germinated when the length of the germ tube exceeded the diameter of the smaller end of the conidium. The percentage of germinated conidia was calculated, and inhibition of *C. xanthochromaticum* germination was determined by the reduction (%) observed in plates with CFF relative to control. The experiment was conducted in a completely randomized design with eight replicates, and data were subjected to t-test (*p* < 0.05).

#### Compatibility of antagonistic bacteria with agricultural pesticides used in macadamia in Brazil

Active ingredients registered for pest and disease control in macadamia were tested with isolates CMAA1920 and CMAA1922. The following products were evaluated at a maximum recommended concentration: abamectin (84 g/L, Vertimec^®^ 84 SC, Syngenta, Basel, Switzerland), acetamiprid (200 g/kg, Mospilan^®^, Nippon Soda, Tokyo, Japan), acibenzolar-S-methyl (500 g/kg, Bion^®^ 500 WG, Syngenta, Basel, Switzerland), azoxystrobin (200 g/L) + difenoconazole (125 g/L) (Amistar Top^®^, Syngenta, Basel, Switzerland), chlorothalonil (500 g/kg, Bravonil^®^ 500 WG, Syngenta, Basel, Switzerland), sulfur (800 g/kg, Kumulus^®^ DF, BASF, Ludwigshafen, Germany), fluxapyroxad (167 g/L) + pyraclostrobin (333 g/L) (Orchestra^®^ SC, BASF, Ludwigshafen, Germany), copper hydroxide (537 g/kg, Kocide^®^ WDG, Mitsui & Co., Tokyo, Japan). Solutions were prepared in sterile distilled water (30 mL) containing twice the maximum recommended concentration and kept under agitation to ensure solubilization. Bacterial antagonists were grown on 523 culture medium at 25 °C for 24 h, suspended in 0.85% NaCl (A₅₄₀ = 0.3), and 30 mL of bacterial suspension was mixed with 30 mL of each product solution. Controls consisted of 0.85% NaCl solution. Duran flasks were incubated on a shaker at 180 rpm at 25 °C. Samples were collected at 1, 6, and 24 h, serially diluted (1:10), plated (100 µL) onto 523 culture medium, and incubated at 25 °C for 24 h. Colony-forming units (CFU/mL) were then counted. Data were log-transformed (ln CFU/mL), analyzed by ANOVA, and compared using Tukey’s test (*p* < 0.05) in R software version 4.3.2 (R Core Team, Vienna, Austria).

## Results

### Initial screening

A total of 104 isolates were obtained from macadamia racemes. Of these, 57 were recovered from reproductive whorls (carpels and stamens) and 47 from vegetative whorls (rachis, peduncle, and petals).

In the antibiosis assay using culture medium diffusion, 43 isolates exhibited inhibitory effects against *C. xanthochromaticum*, with 18 originating from the reproductive whorl and 25 from the vegetative whorls. These 43 isolates were subsequently used in the detached raceme assay.

In the detached raceme assays, twelve isolates reduced the incidence of racemes with *C. xanthochromaticum*: F24, F13, F32, F53, R6, F9, F25, F46, F5, R7, CMAA1920, and CMAA1922. Ten isolates were able to reduce pathogen sporulation: F45, R6, F9, CMAA1920, R20, R32, R37, R38, R39, and CMAA1922 (Table 1). Due to their consistency and higher efficiency in reducing both the incidence and sporulation, isolates CMA1920 and CMAA1922 were selected for subsequent assays.

**Table 1:** Incidence and sporulation of *C. xanthochromaticum* in detached racemes exposed to bacterial isolates

Assay 1			Assay 2		
Treatments	Incidence %*	Sporulation (conidia ml^− 1^)**	Treatments	Incidence %	Sporulation (conidia ml^− 1^)**
F40	98.5^a^	1.34 × 10^6a^	Pathogen-only	100^a^	3.18 × 10^6a^
Pathogen-only	99^a^	3.11 × 10^6a^	R12	100^a^	1.12 × 10^6a^
F6	99^a^	1.28 × 10^6a^	R20	100^a^	3.08 × 10^5b^
F42	98^a^	1.65 × 10^6a^	R21	100^a^	1.71 × 10^6a^
R5	98^a^	3.29 × 10^6a^	R22	100^a^	1.03 × 10^6a^
F27	98^a^	1.23 × 10^6a^	R26	100^a^	1.99 × 10^6a^
F28	96.5^a^	1.63 × 10^6a^	R28	100^a^	1.25 × 10^6a^
F47	95^a^	1.85 × 10^6a^	R32	100^a^	4.42 × 10^5b^
F45	95^a^	4.67 × 10^5b^	R33	100^a^	1.03 × 10^6a^
F26	94^a^	3.45 × 10^6a^	R34	100^a^	7.83 × 10^5a^
F30	94.5^a^	2.20 × 10^6a^	R35	100^a^	1.59 × 10^6a^
F24	93.5^b^	4.45 × 10^6a^	R36	100^a^	1.23 × 10^6a^
F13	93^b^	1.73 × 10^6a^	R37	100^a^	1.63 × 10^5b^
F32	92.5^b^	4.30 × 10^6a^	R38	100^a^	1.42 × 10^5b^
F53	88.5^b^	1.67 × 10^6a^	R39	100^a^	2.58 × 10^5b^
R6	86^b^	1.33 × 10^6b^	R43	100^a^	9.17 × 10^5a^
F9	87^b^	3.67 × 10^5b^	R45	100^a^	7.83 × 10^5a^
F25	85.5^b^	1.31 × 10^6a^	Uninoculated control	99^a^	1.33 × 10^6a^
F46	83^b^	2.25 × 10^6a^	R10	99^a^	1.13 × 10^6a^
Uninoculated control	81^b^	3.99 × 10^6a^	R30	99^a^	6.42 × 10^5a^
F5	80.5^b^	1.63 × 10^6a^	R15	98^a^	7.00 × 10^5a^
R7	80^b^	2.90 × 10^6a^	R25	98^a^	1.08 × 10^6a^
CMAA1920	69.5^b^	2.33 × 10^5b^	R41	98^a^	7.83 × 10^5a^
			CMAA1922	90^b^	2.25 × 10^5b^
CV (%)	22.29	7.32	CV (%)	2.78	6.97

Means followed by the same letter within a column do not differ from each other according to the Scott-Knott test (*p* < 0.05). Data were transformed using *arc-sine(x) and **log(x) for statistical analyses. pathogen-only (racemes inoculated with *C. xanthochromaticum*); uninoculated control (racemes sprayed with sterile distilled water)

### Identification of antagonists

**Fig. 1:**
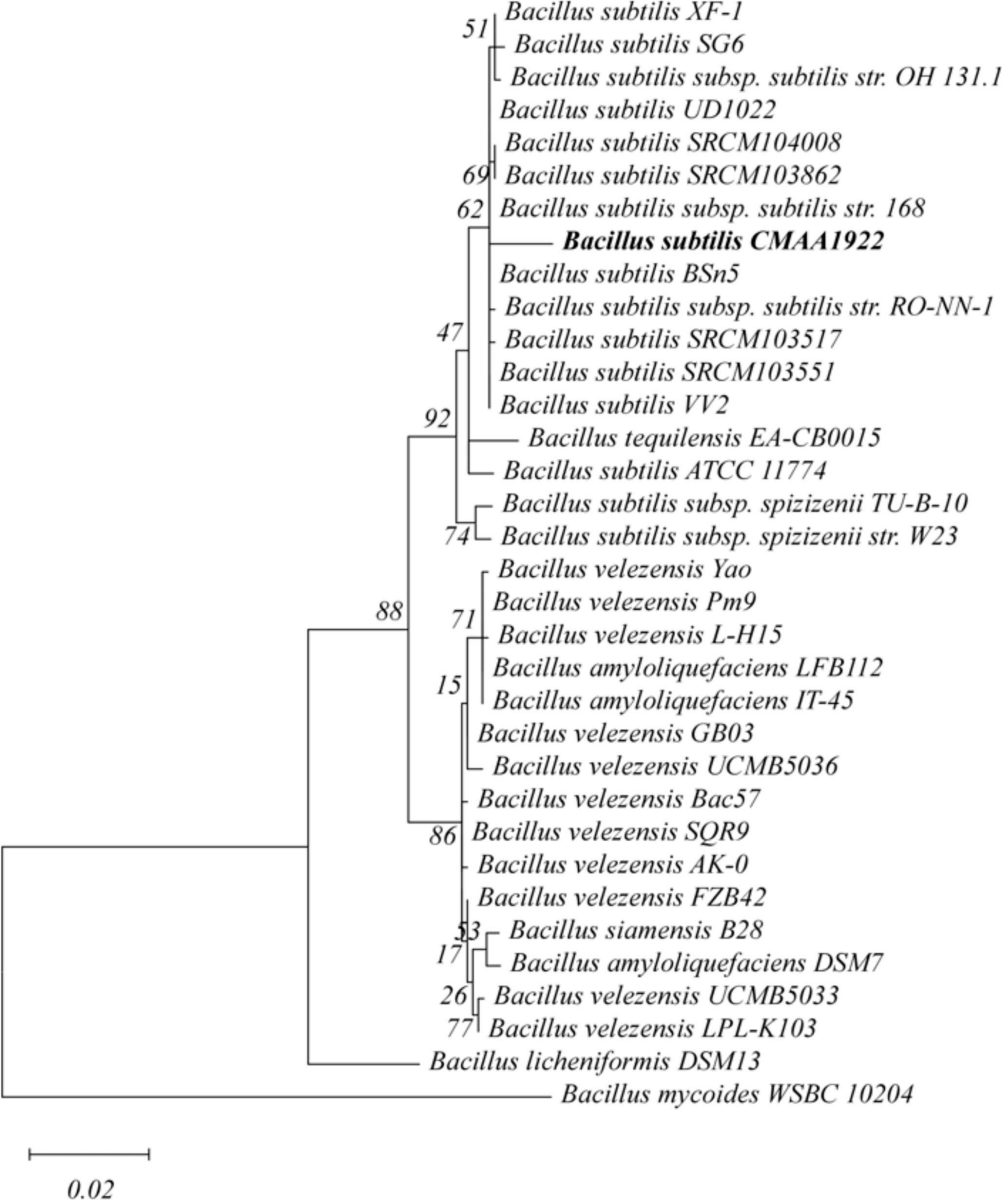
Phylogenetic tree of strain CMAA1922 based on concatenated 16 S rDNA and *tuf* gene sequences. The tree was constructed using the Maximum Likelihood method. Genetic distances were calculated with Kimura’s two-parameter model + G. *Bacillus mycoides* WSBC 10,204 was included as outgroup. Bootstrap values (1,000 replicates) are shown next to the nodes. Scale bar: 0.02 substitutions per nucleotide position

Phylogenetic analysis based on concatenated 16 S rDNA and *tuf* gene sequences revealed that isolate CMAA1922 clustered closely with *Bacillus subtilis* (Fig. 1). The 16 S rDNA and *tuf* sequences were deposited in GenBank under accession numbers PP128333 and PV392971.

**Fig. 2:**
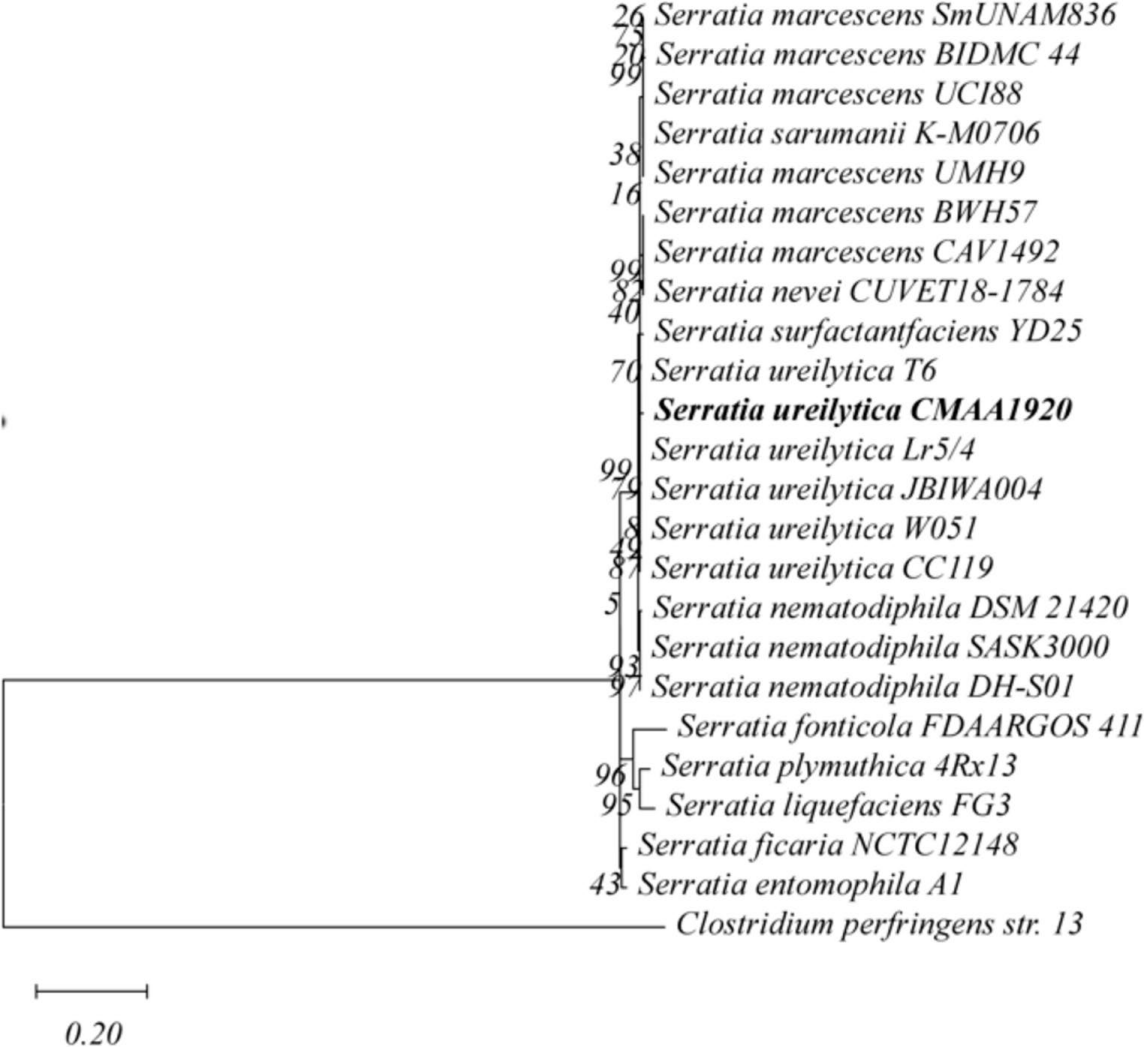
Phylogenetic tree of strain CMAA1920 based on concatenated 16 S rDNA, *gyrB*, and *rpoB* gene sequences. The tree was constructed using the Maximum Likelihood method. Genetic distances were calculated with Kimura’s two-parameter model + G + I. *Clostridium perfringens* str. 13 was included as outgroup. Bootstrap values (1,000 replicates) are shown next to the nodes. Scale bar: 0.02 substitutions per nucleotide position

Phylogenetic analysis based on concatenated 16 S rDNA, *gyrB*, and *rpoB* gene sequences revealed that strain CMAA1920 clustered closely with *Serratia ureilytica* (Fig. 2). The 16 S rDNA, *gyrB*, and *rpoB* sequences of this strain were deposited in GenBank under accession numbers PP128358, PV537013, and PV537014. Furthermore, the strain exhibited non-pigmented colonies and the ability to utilize urea, adonitol, D-melibiose, D-sorbitol, D-xylose, D, L-Alanine, L-Proline, L -Ornithine, L -Threonine, D, L-Serine but not L-Rhamnose and D-Arabinose as sole carbon source, which is consistent with the description of this species (Bhadra et al. 2005).

Both isolates were deposited in the Culture Collection of Microorganisms of Environmental and Agricultural Importance (CMAA) at Embrapa Meio Ambiente, under the voucher codes *Serratia ureilytica* CMAA1920 and *Bacillus subtilis* CMAA1922.

### Field trial

#### Trial 1

**Fig. 3:**
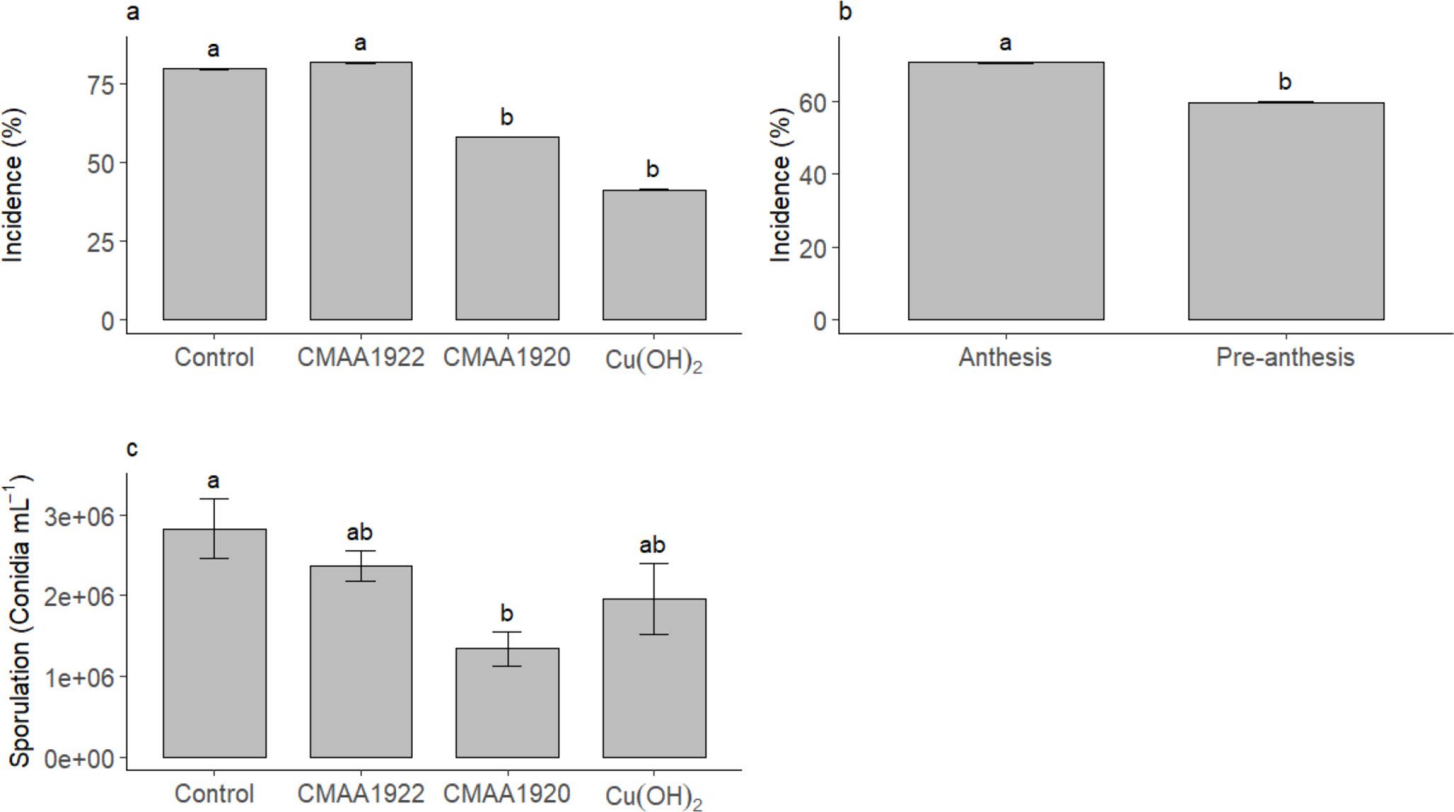
Incidence and sporulation of *C. xanthochromaticum* on macadamia (*Macadamia integrifolia*) racemes. **A**. Incidence on racemes treated with CMAA1920, CMAA1922, and fungicide. **B**. Incidence at different flowering stages. **C**. Sporulation on racemes treated with CMAA1920, CMAA1922, and fungicide. Means followed by the same letter do not differ from each other (Tukey *p* < 0.05). Cu(OH)₂ (commercial copper hydroxide-based product used as the standard treatment, following the manufacturer’s recommendation). Data are means ± standard error (*n* = 18)

In the first trial, no significant interaction between factors was observed. The strain *Serratia ureilytica* CMAA1920 reduced the incidence of racemes with *C. xanthochromaticum* under field conditions, performing similarly to the fungicide. Compared to the control treatment, incidence was reduced by 27.04% and 48.14% when CMAA1920 and the fungicide were applied, respectively. The isolate *Bacillus subtilis* CMAA1922 did not significantly reduce the incidence of racemes with *C. xanthochromaticum*, performing similarly to the control (Fig. 3a). The highest incidence of *C. xanthochromaticum* was observed during anthesis, when flowers were fully open (Fig. 3b), whereas a significant reduction in pathogen incidence was observed at the pre-anthesis stage. For pathogen sporulation on racemes, CMAA1920 reduced *C. xanthochromaticum* sporulation compared to the control, performing similarly to CMAA1922 and the fungicide (Fig. 3c). CMAA1920 reduced pathogen sporulation by 52.65%, the fungicide by 30.74%, and CMAA1922 by 16.25%. Although sporulation was initially recorded for different flowering stages, the statistical analysis did not detect significant differences among stages, and therefore sporulation results are presented only at the whole-raceme level. Thus, among the bacterial strains, CMAA1920 showed the highest efficiency in controlling raceme blight.

#### Trial 2

**Fig. 4:**
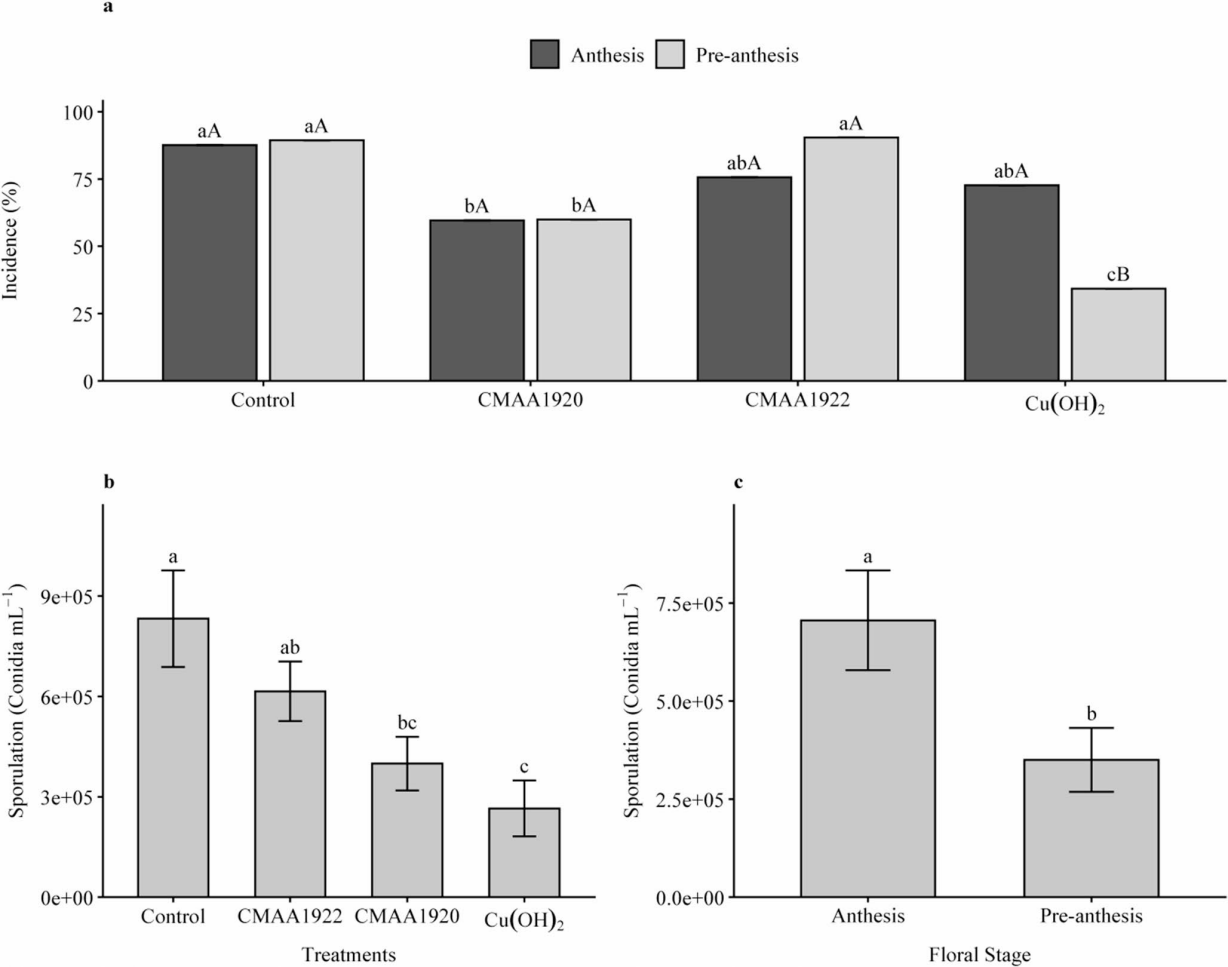
Incidence and *Cladosporium xanthochromaticum* sporulation on macadamia (*Macadamia integrifolia*) racemes. **(A)** Incidence on racemes treated with CMAA1920, CMAA1922, and fungicide at different flowering stages. Means followed by the same lowercase letter within each flowering stage (to compare treatments) or the same uppercase letter within each treatment (to compare flowering stages) do not differ significantly according to the Tukey test (*p* < 0.05). **(B)** Sporulation on racemes treated with CMAA1920, CMAA1922, and fungicide. **(C)** Sporulation at different flowering stages. Means followed by the same letter do not differ significantly (*p* < 0.05). Cu(OH)_2_: commercial copper hydroxide–based product; Data represent means ± standard error (*n* = 18)

A significant interaction (*p* < 0.001) was observed for the incidence of racemes with *C. xanthochromaticum* as a function of the treatment applied and the flowering stage (Fig. 4a). Strain CMAA1920 reduced the incidence compared to the other treatments, regardless of the flowering stage, although it performed similarly to CMAA1922 and the fungicide at anthesis. The greatest reduction in incidence was observed in racemes treated with fungicide at the pre-anthesis stage. Isolate CMAA1922 showed a slight ability to reduce the incidence of *C. xanthochromaticum* when applied at anthesis, performing comparably to strain CMAA1920 and the fungicide at this stage; however, it did not differ from the control treatment.

At anthesis, the greatest reduction in disease incidence was observed with strain CMAA1920 (35.38%), followed by fungicide (17%), and isolate CMAA1922 (13.69%). At pre-anthesis, the greatest reduction in incidence was achieved with fungicide (61.74%), followed by strain CMAA1920 (32.9%).

Sporulation data did not show significant interaction. Among treated racemes, the lowest *C. xanthochromaticum* sporulation was observed in the treatment with fungicide, which reduced sporulation (68.15%), followed by isolate CMAA1920 (52.04%). Strain CMAA1922 showed a numerical reduction in sporulation (26.08%), although it did not significantly differ from the control (*p* > 0.05) (Fig. 4b). Regarding flowering stage, sporulation was lower at the pre-anthesis stage (Fig. 4c). Overall, CMAA1920 was the most effective bacterial isolate in controlling the disease.

### Identification and characterization of the control mechanisms of *Cladosporium xanthochromaticum* by *Bacillus* subtilis and *Serratia ureilytica*

#### Nutritional similarity

Strains CMAA1920 and CMAA1922, together with the phytopathogen CMAA1835, utilized 24 common carbon sources: Dextrin, Glycogen, L-Fucose, Gentiobiose, *m*-Inositol, α-D-Lactose, Lactulose, D-Melibiose, D-Raffinose, Turanose, Xylitol, D-Galacturonic Acid, α-Ketoglutaric Acid, L-Malic Acid, D-Malic Acid, D-Saccharic Acid, Succinic Acid, Glucuronamide, L-Alanyl-Glycine, L-Asparagine, L-Glutamic Acid, Glycyl-L-Glutamic Acid, L-Ornithine, and Glucose-1-Phosphate.

CMAA1922 metabolized eight additional sources: Mannan, Inulin, α-Methyl-D-Glucoside, α-Methyl-D-Mannoside, Sedoheptulosan, α-Hydroxybutyric Acid, γ-Hydroxybutyric Acid, and Lactamide, whereas CMAA1920 metabolized 21 unique compounds: N-Acetyl-D-Galactosamine, N-Acetyl-L-Glutamic Acid, Methyl Pyruvate, Mono-Methyl-Succinate, Cis-Aconitic Acid, Formic Acid, D-Glucosaminic Acid, α-Ketobutyric Acid, Pyruvic Acid, L-Alaninamide, Glycyl-L-Aspartic Acid, Hydroxy-L-Proline, D-Serine, D,L-Carnitine, Urocanic Acid, Inosine, 2’-Deoxyadenosine, Thymidine, Phenylethylamine, and D, L-α-Glycerol Phosphate. CMAA1835 metabolized 13 specific substrates: Alaninamide, Amygdalin, D-Arabinose, L-Sorbose, Maltitol, Palatinose, Fumaric Acid, D-Glucosamine, 2-Keto-D-Gluconic Acid, D-Lactic Acid Methyl Ester, Succinic Acid Mono-Methyl Ester, Succinamic Acid, and L-Pyroglutamic Acid. In total, CMAA1920 metabolized 99 carbon sources, CMAA1922 47, and CMAA1835 86.

Isolate CMAA1920 metabolized 76% of all carbon source compounds, whereas CMAA1922 utilized only 36% of the unique carbon sources. When analyzed by class, CMAA1920 showed greater utilization of amino acids, organic acids, alcohols, amides, amines, and other categories compared with CMAA1835. CMAA1920 also metabolized more sources than CMAA1922, utilizing 52.5% more carbon sources (Table 2).

**Table 2:** Number of compounds utilized as sole carbon sources by the selected antagonistic isolates and *Cladosporium xanthochromaticum*

Isolate	Carb.	Amino.	Org. Ac.	Alcohols	Amid.	Others
CMAA1920	32	16	27	4	6	14
CMAA1922	20	6	12	1	2	6
CMAA1835	38	12	20	2	5	10
Sources	44	18	37	4	9	18

Total = all 130 unique carbon sources, including unclassified compounds; *Carb*  carbohydrates, *Amino *amino acids, *Org. Ac.*  organic acids, *Ami *amides and amines, Sources = number of carbon source classes tested. CMAA1920 (*Serratia ureilytica*), CMAA1922 (*Bacillus subtilis*), and CMAA1835 (*C*. *xanthochromaticum*)

Considering all carbon sources, no significant niche overlap index (NOI) was detected for CMAA1920 or CMAA1922 over CMAA1835 (Table 3). A significant NOI was observed only for isolate CMAA1920 for amino acids and alcohols. CMAA1835 showed a significant NOI only for carbohydrates in relation to the antagonist CMAA1920.

**Table 3:** Niche overlap indexes (NOIs) for antagonistic bacteria paired with *Cladosporium xanthochromaticum*, based on compound utilization data

Isolate	Total		Carb.		Amino.		Org. Ac.		Alcohols		Amid.			Others	
	NOI_ant_	NOI_cx_	NOI_ant_	NOI_cx_	NOI_ant_	NOI_cx_	NOI_ant_	NOI_cx_	NOI_ant_	NOI_cx_	NOI_ant_	NOI_cx_	NOI_ant_		NOI_cx_
CMAA1920	0.8	0.69	0.8	0.94	0.9	0.69	0.7	0.52	1	0.5	0.6	0.5	0.8		0.57
CMAA1922	0.34	0.62	0.39	0.75	0.4	0.83	0.3	0.5	-	-	0.2	0.5	0.2		0.33

NOI_ant_ represents the niche overlap of antagonistic bacteria over *C. xanthochromaticum*. NOI_cx_ represents the niche overlap index of *C. xanthochromaticum* over antagonistic bacteria. NOIs were derived from substrate utilization data generated using Biolog GP2 and GN2 microplates. Total = all 95 unique carbon sources, including unclassified compounds; *Carb* carbohydrates, *Amino* amino acids, *Org. Ac.*  organic acids, *Ami *amides and amines, NOIs > 0.9 indicate significant niche overlap, whereas NOIs < 0.9 indicate nonsignificant niche overlap

#### Antibiosis by volatile compounds

The in vitro assay evaluating the inhibition of *C. xanthochromaticum* mycelial growth by volatile compounds revealed that both CMAA1920 and CMAA1922 isolates suppressed the pathogen’s growth. Inhibition reached 100% for isolate CMAA1920 and 52.79% for isolate CMAA1922.

#### Determination of volatile compounds

The volatile compound (VC) profile revealed a diversity of molecules produced by the isolates. Strains CMAA1920 and the strain CMAA1922 produced four volatile compounds (Table 4). Three VCs (acetoin, dimethyl disulfide, and dimethyl trisulfide) were shared by both strains. Specifically, CMAA1920 produced methyl vinyl ketone, while CMAA1922 produced 2,3-butanedione. The identification of volatile compounds was performed using Agilent MassHunter Unknown Analysis software, applying deconvolution to detect individual components in the sample when the compounds are co-eluted, followed by comparison of the obtained spectra with mass spectrum libraries. This provides a list of components, the deconvolved spectrum of each component, and the matching factor, which indicates the similarity between an acquired mass spectrum and the reference spectrum from the library. To identify the compounds, we used the match factor (CF), where values equal to or greater than 90.0 are considered excellent, values between 80.0 and 90.0 good, between 70.0 and 80.0 reasonable, and less than 60.0 poor. The matching factors for all compounds were considered excellent or good, except for the dimethyl disulfide compound from *Serratia ureilytica* CMAA1920 (match of 69.52). Although this compound has been reported in the literature as being produced by bacteria, its presence in the sample requires further confirmation. The GC-MS data are provided as supplementary material (Figure S1).

**Table 4:** Profile of volatile organic compounds (VOCs) produced by *Serratia ureilytica* CMAA1920 and *Bacillus subtilis* CMAA1922

VOCs name	Retention time (min)	CAS No.	Match
CMAA1920			
Acetoin	5.0772	513-86-0	92.52
Methyl vinyl ketone	5.5543	78-94-4	92.34
Dimethyl disulfide	5.8188	624-92-0	69.52
Dimethyl trisulfide	11.7758	3658-80-8	96.81
CMAA1922			
2,3 butanedione	4.2465	431-03-8	90.36
Acetoin	5.1294	513-86-0	88.26
Dimethyl disulfide	5.8238	624-92-0	85.4
Dimethyl trisulfide	11.75	3658-80-8	92.35

#### Inhibition of conidial germination by cell-free filtrate

The t-test revealed no significant differences between CMAA1920 and CMA1922 in inhibiting *C. xanthochromaticum* conidial germination by the cell-free filtrates. Strain CMAA1920 inhibited 77.31 ± 8.97% of pathogen spore germination, while CMAA1922 inhibited 79.67 ± 13.09%.

#### Compatibility of antagonistic bacteria with pesticides used in macadamia in Brazil

The isolates CMAA1920 and CMAA1922 showed compatibility with almost all active ingredients tested (Table 5). After 24 h, CFU remained stable and similar to control, except in the treatment with copper hydroxide, which significantly reduced the CFUs of isolate CMAA1920, indicating incompatibility with this compound.

**Table 5:** Colony-forming unit (CFU mL⁻¹) counts of *Serratia ureilytica* (CMAA1920) and *Bacillus subtilis* (CMAA1922) in compatibility tests with active ingredients used in macadamia

Active ingredient	CMAA1920			CMAA1922		
	Time (h)			Time (h)		
	1	6	24	1	6	24
Abamectin (84 g/L)	19.76aA	19.30aB	19.21aB	15.8abcA	15.9aA	15.1bB
Acetamiprid (200 g/kg)	19.02bA	19.25aA	19.20aA	15.2bcB	15.3abB	16.7aA
Acibenzolar-S-methyl (500 g/kg)	18.94bA	18.97aA	19.12aA	16.2aAB	15.7abB	16.4aA
Azoxystrobin (200 g/L) + Difenoconazole (125 g/L)	19.58aA	19.12aB	19.25aAB	15.0cB	14.8bcB	16.5aA
Chlorothalonil (500 g/L)	19.59aA	19.18aB	19.32aAB	15.2cB	14.2cC	16.9aA
Control	19.61aA	19.14aB	19.27aAB	16.2abA	15.7abA	16.3aA
Sulfur (800 g/kg)	18.86bA	17.66bC	18.12bB	15.1cB	15.4abB	16.5aA
Fluxapyroxad (167 g/L) + Pyraclostrobin (333 g/L)	19.27abA	19.28aA	19.30aA	15.8abcA	15.8aA	16.1aA
Copper hydroxide (537 g/kg)	13.14cA	9.58cB	3.27cC	14.9cB	15.8aA	14.8bB

Means followed by the same letter, lowercase in the column and uppercase in the row, do not differ from each other (Tukey’s test, *p* < 0.05). Control = sterilized distilled water

## Discussion

Our results demonstrate that bacteria obtained from the macadamia anthosphere have potential as biocontrol agents against raceme blight caused by *C. xanthochromaticum*. Detached raceme assays identified several inhibitory isolates, leading to the selection of two promising strains, *Serratia ureilytica* CMAA1920 and *Bacillus subtilis* CMAA1922, which were subsequently validated under field conditions. To our knowledge, this is the first report addressing the use of antagonistic bacteria for the control of Cladosporium raceme blight in macadamia. Previous studies on biological control in macadamia have mainly focused on stem or radicular pathogens (Li et al. 2022; Velázquez and Ocampos 2016), highlighting the novelty of our findings.

*Bacillus subtilis* is a well-known biocontrol agent against several plant diseases. Its modes of action include antibiosis, competition for space and nutrients, and induction of host defense mechanisms (Belete et al. 2021; Etesami et al. 2023; Van Toor et al. 2005; Zitter et al. 2005). In contrast, reports on *Serratia ureilytica* as a biocontrol agent are scarce, being limited to its nematicidal activity (Wong-Villarreal et al. 2021) and suppression of tomato damping-off caused by *Pythium cryptoirregulare* (Abreo et al. 2021). The present study, therefore, expands the knowledge on *S. ureilytica* by demonstrating its potential role in controlling the major flower disease of macadamia in Brazil.

Both bacterial isolates, CMAA1920 and CMAA1922, demonstrated potential for controlling *C. xanthochromaticum* under field conditions. CMAA1920 exhibited similar efficiency to the commercial fungicide Supera^®^, effectively reducing the disease incidence and pathogen sporulation, indicating that it could serve as a biological alternative to chemical control. CMAA1922 was particularly effective in reducing pathogen sporulation, which could impact the disease epidemics by lowering the inoculum density.

Regarding floral development, lower incidence and sporulation were generally observed at the pre-anthesis stage. *Cladosporium* spp. can colonize macadamia racemes at any developmental stage and is considered a latent pathogen, as it may remain endophytic until favorable conditions trigger disease progression (Prasannath et al. 2022a, 2023; Silva et al. 2024; Sosso et al. 2021). Therefore, preventive treatments during pre-anthesis may be crucial to limit disease development in the field, especially when combined with monitoring of environmental conditions favorable to pathogen growth.

For raceme blight caused by *Cladosporium* spp., reducing sporulation is particularly important since these pathogens are characterized by colonization of floral structures with prolific spore production, which serves as inoculum source for new infections (Prasannath et al. 2022a, b). Both CMAA1920 and CMAA1922 effectively reduced *C. xanthochromaticum* sporulation, indicating that these strains could mitigate disease damage and interfere with pathogen dissemination in macadamia orchards.

Both CMAA1920 and CMAA1922 isolates exhibited clear antagonistic activity against *C. xanthochromaticum*, acting through multiple mechanisms, including the production of antifungal molecules that inhibit mycelial growth and spore germination, the synthesis of diffusible and volatile compounds. Wong-Villarreal et al. (2021) demonstrated that *S. ureilytica* UTS strain possesses genes capable of producing chitinases, while bacteria of the genus *Bacillus* are also known to produce a wide of antimicrobial compounds involved in biocontrol activities (Etesami et al. 2023). However, in the present study, neither isolate showed the ability to produce chitinase or β-1,3-glucanase, indicating that the inhibition of mycelial growth and spore germination is not related to these lytic enzymes.

On the other hand, only CMAA1920 demonstrated high metabolic versatility, utilizing a wide range of carbon sources, including amino acids and alcohols. Its efficiency in metabolizing 89% amino acids and 100% of alcohols of the tested substrates, compared to 67% and 50%, respectively used by *C. xanthochromaticum*, suggests a competitive exclusion mechanism that limits resources available to the pathogen. In contrast, CMAA1922 demonstrated a lower capacity for nutrient utilization, which may explain its inefficiency in reducing pathogen incidence on detached racemes and under field conditions. A study with *C*. *herbarum* corroborated the idea that fungus development is strongly influenced by the availability of nutrients. In the presence of pollen, 7,700 spores per cm² were observed, whereas only 960 spores per cm² developed in its absence (Fokkema 1968). Given that pollen provides carbon and nitrogen sources (Roulston and Cane 2000), competition for these resources interferes with inoculum potential prior to host penetration. These aspects indicate that, in this pathosystem, the ability to employ multiple mechanisms of action is crucial for effective disease control.

Our results indicate that the VOCs produced by these strains acted as important inhibitory agents against pathogen development. The antifungal activity of VOCs has been associated with their ability to disrupt cell wall integrity, induce oxidative stress in fungal cells, and alter plasma membrane structure leading to leakage of intracellular content (Zhao et al. 2022). Herrington et al. (1987) demonstrated the fungistatic effect of methyl vinyl ketone, produced by Streptomyces griseoruber, in the inhibition of C. cladosporioides spore germination. In our study, CMAA1920 also produced this volatile compound, suggesting its involvement in the capacity to inhibit pathogen development. Dimethyl disulfide has also been reported as a product of S. ureilytica ILBB 145, with activity against the mycelial growth of the oomycete Pythium cryptoirregulare (Abreo et al. 2021), and antifungal effect against Phyllosticta citricarpa (Toffano et al. 2017) and Penicillium italicum in combination with dimethyl trisulfide (Wang et al. 2021). Moreover, 2,3-butanedione and acetoin are reported to promote plant growth (Ryu et al. 2003), act as elicitors of defense responses against phytobacteria, and cause physiological damage in bacterial cells such as Ralstonia solanacearum (Tahir et al. 2017). However, in the present study the antifungal activity of these individual VOCs was not validated using pure compounds, and their specific contribution to pathogen inhibition therefore remains inferred rather than experimentally confirmed. Further work using bioassays with isolated VOCs will be required to disentangle their relative roles in the observed inhibitory effects. Our results corroborate the potential of CMAA1920 and CMAA1922 as producers of volatile compounds with inhibitory activity against pathogens, offering new perspectives for disease control in macadamia.

The results also show that strains CMAA1922 and CMAA1920 were compatible with the main active ingredients used for macadamia (Table 5), except for CMAA1920, which was sensitive to copper hydroxide. This compatibility supports the adoption of these antagonists as components of integrated disease management, also enabling simultaneous control of *C. xanthochromaticum* through biocontrol agents and of floral pests through pesticides. Such practices are particularly relevant in macadamia, where the simultaneous occurrence of pathogens and invertebrate pests requires integrated solutions. Several studies have confirmed the compatibility of biocontrol agents with pesticides, especially *Bacillus* spp., and have further highlighted cases of synergistic interactions, where their combined use enhances disease control efficacy (Ons et al. 2020; Peng et al. 2014, 2017; Su et al. 2022). Although CMAA1920 is sensitive to copper hydroxide, it was fully compatible with the following fungicide groups: chlorothalonil, strobilurins, triazoles, and carboxamides. Therefore, CMAA1920 can be applied in combination with these fungicides without interfering with its development.

Although *S. ureilytica* CMAA1920 showed promising efficacy as a biological control agent, members of the *S. ureilytica* complex have been reported as opportunistic pathogens in vulnerable human populations. For this reason, potential exposure routes, including contact by farm workers, environmental dissemination in orchards, and possible indirect exposure through nut contamination, should be carefully considered before large-scale deployment. In the present study, safety aspects were not experimentally evaluated, and the use of CMAA1920 should therefore be regarded as preliminary and subject to further risk assessment. Future work should address toxicity and pathogenicity tests, as well as monitoring of bacterial persistence on plant surfaces and nuts, to ensure that disease control benefits are achieved without compromising human or environmental health.

## Conclusion

This study demonstrated that the biocontrol agents *Serratia ureilytica* CMAA1920 and *Bacillus subtilis* CMAA1922 effectively control Cladosporium raceme blight in macadamia under field conditions. The strains exhibited multiple mechanisms of pathogen suppression, primarily through the production of inhibitory compounds. Furthermore, both agents showed compatibility with the main pesticides used in macadamia production in Brazil, enabling their combined applications within integrated disease management programs.

## Future perspectives

Future studies should focus on disentangling the specific contribution of individual VOCs produced by *S. ureilytica* CMAA1920 and *B. subtilis* CMAA1922 to disease control and the major non-volatile compounds produced by these strains. Furthermore, elucidate the ability of these antagonists to control other pathogens that cause macadamia raceme blight, is valuable for determining the breadth of their biocontrol spectrum. Another key research direction is the optimization of fermentation conditions for *S. ureilytica* CMAA1920 to support the development of scalable formulations for field application. Together, these efforts will help refine strain selection, improve the robustness of biological control, and facilitate the integration of these bacteria into macadamia raceme blight management programs.

## Supplementary Information

Below is the link to the electronic supplementary material.

Supplementary Material 1

Supplementary Material 2

Supplementary Material 3

## Data Availability

Data will be made available on request.
